# Developmental exposure to low doses of dichlorodiphenyltrichloroethane impairs proliferative response of thymic lymphocytes to Concanavalin A in rats

**DOI:** 10.1016/j.heliyon.2020.e03608

**Published:** 2020-03-17

**Authors:** Nataliya V. Yaglova, Elina S. Tsomartova, Sergey S. Obernikhin, Marina Y. Ivanova, Elizaveta V. Chereshneva, Svetlana G. Muhamedova, Tatiana A. Lomanovskaya, Valentin V. Yaglov

**Affiliations:** a Laboratory of Endocrine System Development, Federal State Budgetary Institution Research Institute of Human Morphology, Moscow, Russia; b Department of Histology, Cytology, and Embryology, Federal State Funded Educational Institution of Higher Education I.M. Sechenov First Moscow State Medical University, Moscow, Russia

**Keywords:** Thymus, Proliferative response, Concanavalin A, Development, Thymic lymphocytes, Endocrine disrupter, Dichlorodiphenyltrichloroethane, Cell differentiation, Immunology, Developmental biology, Toxicology, Immune system

## Abstract

The aim of the research was to study formation of thymic lymphocytes proliferative response to T cell mitogen Concanavalin A in 7, 42, and 70 days-old male Wistar rats developmentally exposed to low doses of endocrine disruptor dichlorodiphenyltrichloroethane (2.90 ± 0.13 μg/kg body weight). The thymus of the exposed rats did not show morphological abnormalities. Exposure to the endocrine disrupter was found to alter age-dependent changes of thymic lymphocyte proliferative activity and attenuate proliferative response to Concanavalin A in puberty and adulthood. Insufficient response to mitogen was mediated by higher content of actively proliferating Ki-67-positive lymphoblasts compared to the control values. Insufficient proliferative response to mitogen in developmentally exposed to the endocrine disruptor rats may provide higher risk of impaired cellular immune reactions.

## 1 Introduction

Dichlorodiphenyltrichloroethane (DDT) is considered one of the most wide-spread persistent organic pollutant on the planet [1, 2]. Pervasion of the environment by DDT and its metabolites is a result of massive use as a pesticide in the 20^th^ century. DDT was banned in many developed countries, but in developing countries it is still used for disease vector control. Continued extensive use as insecticide for malaria control in Africa, Asia and Latin America and long half-life provide further worldwide contamination of environment by DDT and its metabolites [3]. DDT remains a widespread food contaminant that is why the main source of low-dose DDT exposure is food products. The danger of low-dose exposure is associated with high lipophylicity and low molecular weight of DDT. DDT and its metabolites easily penetrate blood-tissue barriers and accumulate in different inner organs especially in adipose tissue [4]. Transplacental transfer of DDT has been found to impair development of fetal endocrine and reproductive system [5, 6]. DDT is also known to excrete with breast milk, and breast-feeding is still a considerable route of exposure in infants since levels of DDT remain high in breast milk recent years [7, 8].

The hazard of low-dose exposure to DDT is associated with its identified endocrine disrupting properties [2, 9]. Growing number of scientific reports demonstrate that increase in incidence of immune system-associated diseases parallels with world-wide increasing usage of endocrine disrupting chemicals [10, 11, 12, 13]. Disruption of endocrine system development and endocrine regulation of prenatal and postnatal development as an origin of immunological disorders in infants and adults is gaining more scientific evidence [14]. Impact of low-dose developmental exposure to DDT and metabolites on thymus organogenesis and function is still unclear. Our previous investigations revealed activation of thymic cells apoptosis and accelerated involution of thymus after chronic exposure of adult rats to low doses of DDT [15] and made a background for further study of DDT exposure during prenatal and postnatal development. In this study, proliferation of thymic lymphocytes and proliferative response to T-cell mitogen Concanavalin A during ontogeny was assessed. Development of proliferative response is crucial for adaptive immunity because blastogenic transformation and proliferation of T-cells after antigenic stimulation provide cell mediated reactions of immune defense.

## 2 Materials and methods

### 2.1 Chemical

2-(2-chlorophenyl)-2-(4-chlorophenyl)-1,1,1-trichloroethane (o,p′-DDT) isomer of DDT was used because of higher solubility in water (Sigma-Aldrich). Water solution with concentration of DDT 20 μg/l was prepared for substitution of tap water as a route of exposure of animals.

### 2.2 Laboratory animals and experimental design

Wistar rats were purchased from Scientific center of biomedical technologies of Federal Medical and Biological Agency of Russia. The rats were housed at +22–23 °C with a 12/12-hr light-dark cycle and given a pelleted standard chow ad libitum.

The female rats received solution of o,p-DDT 20 μg/l (“Sigma-Aldrich”, USA) ad libitum instead of tap water since mating during pregnancy and lactation. After weaning the progeny of the rat dams received the same solution of o,p-DDT during postnatal development. The absence of DDT and its metabolites and related organochlorine compounds in pelleted standart chow and tap water was confirmed by high performance liquid chromatography and mass spectrometry performed in Federal budgetary institution of public health “Center for Hygiene and Epidemiology in Moscow”. Average daily intake of DDT was calculated as a mean of daily consumed volumes of DDT solution (DDT concentration - 20 μg/l) per kg bw. It was 2.47 ± 0.11 for pregnant dams, 2.69 ± 0.11 for lactating dams, and 2.90 ± 0.13 μg/kg body weight for offspring after weaning. These levels of exposure are consisted with criteria of low-dose exposure for DDT [16, 17]. Only male offspring was enrolled in the investigation because of pronounced sex differences in histophysiology of rat immune system. The male offspring of intact dams was considered as control group. The DDT-exposed rats (n = 36) and the control rats (n = 36) was sacrificed gradually on the 7^th^ (neonatal period), 42^nd^ (puberty) and 70^th^ (adulthood) day of postnatal development by overdosage of zoletil (Virbac Sante Animale). The rats and the excised thymi were weighted. Relative weight of the thymus was calculated and presented as a percent of body mass. Right and left lobes of the thymi were dissected. One lobe was used for histological examination, the other one – for proliferation assay.

Animal experiments were approved by The Ethics committee of the Institute of Human Morphology on the 12 of September 2018 (protocol No. 8). The investigation was performed in accordance with the handling standards and rules of laboratory animals as consistent with “International Guidelines for Biomedical Researches with Animals” (1985), laboratory routine standards in the Russian Federation (Order of Ministry of Healthcare of the Russian Federation dated 19.06.2003 No.267) and “Animal Cruelty Protection Act” dated 1.12.1999, regulations of experimental animal operation approved by Order of Ministry of Healthcare of USSR No.577 dated 12.08.1977.

### 2.3 Primary cell culture

Thymus was homogenized in a 40μm cell strainer and washed with RPMI 1640 cell culture media supplemented with L-glutamine (2 mmol/ml), 10% heat-inactivated fetal calf serum and glutamine. After three cycles of centrifugation and washing thymic lymphocytes were stained with trypane blue to assess viability. Viability of the cells was always more than 95%. Cells were resuspended with complete RPMI 1640 cell culture media (2 × 10^6^ cells/ml) for proliferation *ex tempore* and proliferative response to mitogen assays.

### 2.4 Proliferation *ex tempore* assay

Proliferation *ex tempore* technique [18] allows to evaluate cell proliferative activity close to real proliferation rate *in vivo*. Cell suspension samples were transferred to U-bottom 96-well plate in triplicates (0.1 ml per well). ^3^H-thymidine (Sigma-Aldrich) was added (1μCi per well). The plates were incubated in atmosphere with 5% CO_2_ during 4 h. The cells were harvested onto glass fiber filter paper with cell harvester. ^3^H-thymidine uptake by the cells was estimated by counting of filters with “1209 Rackbeta” liquid scintillation counter (LKB, Austria).

### 2.5 Thymic lymphocyte response to mitogen

Thymus cell suspension samples (0.1 ml) was dispensed into U-bottom 96-well plate. The same volume of complete RPMI 1640 media was added in triplicates to wells containing no mitogen for assessment of background proliferation. An aliquote of 0.1 ml of T-cell mitogen Concanavalin A (Sigma-Aldrich) solution with concentration 5 μg/ml was added to triplicates of each cell sample. Culture was incubated for 72 h at 37 °C in 5% CO_2_. 4 hours before harvesting 1μCi ^3^H-thymidine was added to each well. The cells were harvested and counted as described above. Proliferative response to mitogen was determined using means of triplicate as follows:

Index of proliferation (IP) = ^3^H-thymidine incorporation with Concanavalin A (counts per minute)/^3^H-thymidine incorporation without Concanavalin A (counts per minute).

### 2.6 Histological examination

Thymus was fixed in Bouen solution and after standard histological processing embedded in paraffin. Sections of the thymi were stained with hemotoxylin and eosin. Areas of cortex and medulla were assessed using “Image Scope Color” software (Leica Microsystems Gmbh).

### 2.7 Immunohistochemistry

Ki-67 polyclonal antibodies (Cell Marque) were used for evaluation of subcapsular layer of lymphoblasts. Immunohistochemistry was performed according to manufecturer's recommendations and visualized with «UltraVision LP Detection System» (ThermoScientific). Width of lymphoblast layer was measured using “ImageScope” software (Leica Microsystems Gmbh).

### 2.8 Statistical analysis

The data were processed using Statistica 7.0 software (StatSoft, Inc.). The data were expressed as mean and standard error of mean (M±SEM). Comparison of the groups was performed using one way ANOVA (Duncan test), Student's *t*-test and χ^2^. The differences were significant at *p* < 0.05.

## 3 Results

The control rats demonstrated increase of thymus mass and reduction of relative thymus weight with age (Table 1). The DDT-exposed rats showed age-dependent changes of absolute and relative thymus weight similar to those in the control group. At the age of 42^nd^ days thymus mass was slightly increased compared to the control, but the difference was statistically insufficient (Table 1).

**Table 1:** Age-dependent changes of thymus anatomy and histology in the control and developmentally exposed to low doses of endocrine disrupter DDT rats.

Age	Parameter	Control rats	DDT-exposed rats
7 days	Thymus weight, g	0.30 ± 0.0015	0.30 ± 0.002
	Relative thymus weight, %	0.22 ± 0.01	0.2 ± 0.018
	Cortex area, %	85.72 ± 3.30	85.87 ± 0.23
42 days	Thymus weight, g	0.54 ± 0.045+	0.66 ± 0.05+
	Relative thymus weight, %	0.2 ± 0.01+	0.25 ± 0.019
	Cortex area, %	72.58 ± 0.25+	67.89 ± 2.76+
70 days	Thymus weight, g	0.66 ± 0.01+	0.64 ± 0.02
	Relative thymus weight, %	0.19 ± 0.001	0.18 ± 0.004
	Cortex area, %	73.88 ± 0.91	74.91 ± 1.22

Values are presented as M±SEM. + – p < 0.05 compared to the previous age.

**Figure 1:**
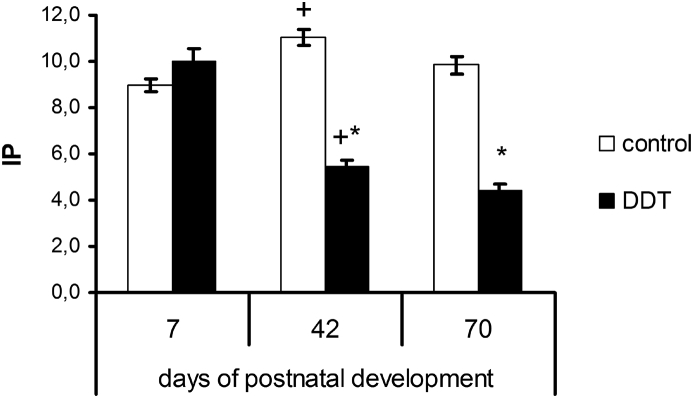
Proliferative response of thymic lymphocytes to Concanavalin A in rats developmentally exposed to low doses of DDT. ∗ – p < 0.05 compared to control, + – p < 0.05 compared to previous age period.

Evaluation of proliferative response to Concanavalin A showed no significant differences on the 7^th^ day between the exposed and the control rats. On the 42^nd^ day index of proliferation in the control rats reached maximal values. On the 42^nd^ and 70^th^ days index of proliferation in DDT-exposed rats decreased compared to the 7^th^ day and was two times lower than in the control rats of the appropriate age (Figure 1).

**Figure 2:**
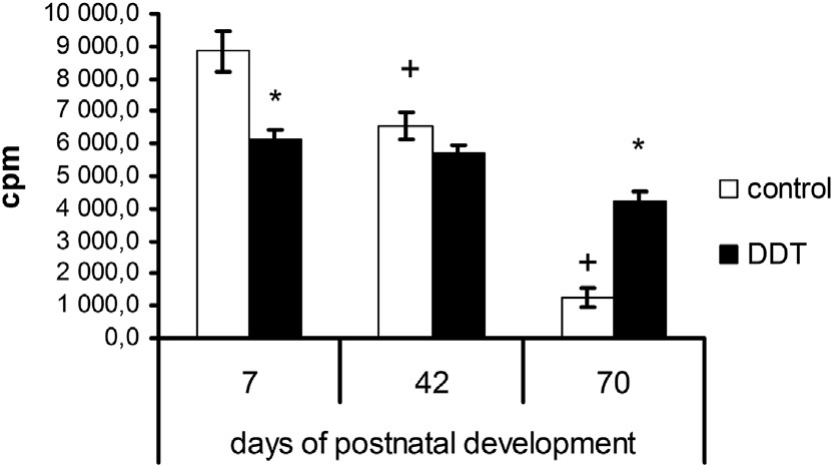
Age-dependent changes of proliferation ex tempore rate of thymic lymphocytes in rats developmentally exposed to low doses of DDT. ∗ – p < 0.05 compared to control, + – p < 0.05 compared to previous age period.

Proliferative activity of thymic lymphocytes in the DDT-exposed rats was diminished on the 7^th^ day, but on the 70^th^ day proliferation of thymic lymphocytes significantly exceeded the control values. Unlike the control the DDT-exposed rats demonstrated constant rate of proliferation on the 7^th^ and 42^nd^ days of postnatal development (Figure 2).

Histological examination of the thymus in the neonatal, pubertal and adult DDT-exposed rats revealed typical structure with distinct cortex and medulla. No statistically significant differences in surface area of the cortex at the 7^th^, 42^nd^, and 70^th^ days of postnatal development between the exposed and control rats was found (Table 1).

**Figure 3:**
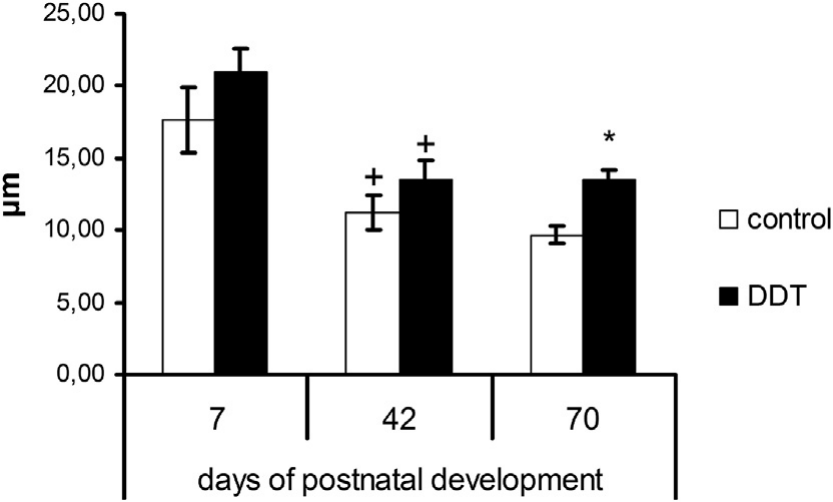
Width of subcapsular layer of lymphoblasts in rats developmentally exposed to low doses of DDT. ∗ – p < 0.05 compared to control, + – p < 0.05 compared to previous age period.

**Figure 4:**
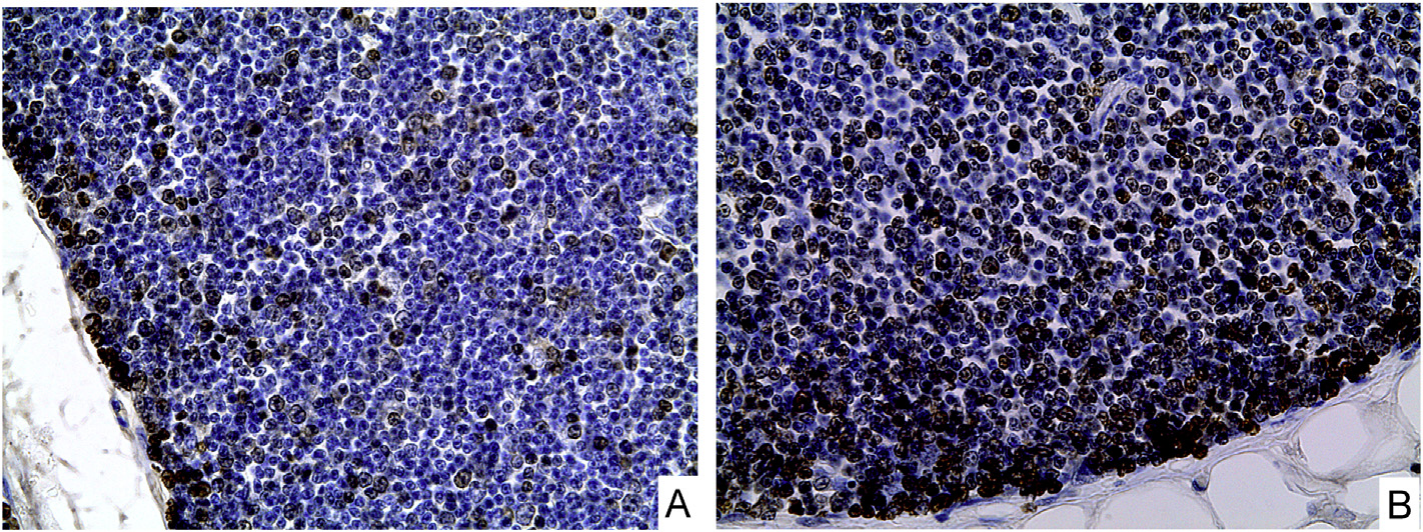
Immunohistochemical evaluation of Ki-67-positive subcapsular lymphoblasts in the thymus of 70-days old rats of the control group (A) and after developmental exposure to low doses of DDT (B). Thymus of the DDT-exposed rats exhibits wider layer of mitotically active lymphoblasts. Nuclei were counter-stained with hematoxylin. 400X.

Immunochemical evaluation of Ki-67 revealed a distinct compact subcapsular layer of mitotically active lymphoblasts in the thymus. In the control rats the layer of lymphoblasts gradually reduced with age (Figures 3, 4a). The DDT-exposed rats exhibited less pronounced age-dependent changes in width of subcapsular layer. Statistically significant enlargement of lymphoblast layer was found on the 42^nd^ and 70^th^ day (Figures 3, 4b).

## 4 Discussion

The ability to respond to mitogenic stimuli by blastogenic transformation and activation of proliferation is acquired by lymphocytes during their maturation [19], and is associated with the appearance of functionally active lectin receptors on the outer membrane of the lymphocyte. In rodents formation of proliferative response of T-cells to mitogen begins in prenatal development, 2–3 days prior to birth [20, 21]. The decrease in proliferation and a magnification of proliferative response of T-cells to mitogen indicate an increase in the number of differentiated lymphocytes in the thymus. It has been shown that proliferative response to mitogen in mice and rats accelerates after birth [19]. Our investigation of proliferative response to Concanavalin A during postnatal development found that it was well-developed in neonatal rats and reached maximal values in puberty. DDT-exposed rats also demonstrated adequate parameters of proliferative response in the neonatal period with its gradual decrease with age. The reason for decreased proliferative response was revealed after assessment of proliferation *ex tempore* which values are similar to proliferation rate of cells *in vivo*. Thymus parenchyma declines with age [22]. It was associated with lower proliferation rate of thymic lymphocytes. In the present study we also observed gradual decrease of proliferation *ex tempore* rates with age in the control animals. DDT-exposed rats exhibited other age-dependent changes of proliferative activity with an initially lower level of cell division, which practically did not decrease in postnatal development. After puberty higher than the control values proliferation *ex tempore* indicated that the cortex of the thymus contained more low-differentiated cells. Histological examination found no significant changes in development of thymic parenchyma, but immunogistochemical evaluation revealed bigger content of proliferating lymphoblasts in the cortex in puberty and adulthood. Increased number of low-differentiated cells provided higher proliferation *ex tempore* rate and lower proliferative response to mitogen.

Our previous studies revealed that exposure of adult rats to low doses of DDT induced apoptosis of thymic and splenic lymphocytes [15, 23], but the present findings demonstrate that low dose-exposure to DDT during ontogeny promotes proliferation and downregulates differentiation of lymphoblasts in the thymus. It suggests that low-differentiated T-cells are less sensitive to endocrine disruption-mediated apoptotic stimuli, and DDT acts as morphogenic disruptor. The most pronounced disorders we found during and after pubertal period when lymphoid cells in rats are known to acquire immune competence [24, 25]. These data suggest impaired immune response in adults and higher susceptibility to infectious diseases, because low proliferative response of lymphocytes to mitogens has been found to be a predictor of higher severity of infectious diseases and poor prognosis for patients [26, 27]. The exact mechanisms by which DDT exerts the disregulatory effects on immune system is not fully understood and require further investigations. We suppose that appeared in puberty disorders in proliferation and differentiation of thymocytes might be provoked both by alterations in programme of thymus development, direct disruptive action on thymic lymphocytes during postnatal development, and impaired hormonal regulation of thymus development since DDT has been found to produce multiple disorders in endocrine system [28, 29, 30].

## 5 Conclusion

The present study reveals that formation of proliferative response of thymic lymphocytes to T-cell mitogen in rats, prenatally and postnatally exposed to endocrine disrupter DDT, inhibits during postnatal development. Attenuated proliferative response is associated with lower differentiated state and proliferation activity of the thymic lymphocytes. The data obtained suggest putative impairment of cellular immune response in adults as a result of developmental exposure to low doses of the endocrine disrupter DDT.

## Declarations

### Author contribution statement

N.V. Yaglova, S.S. Obernikhin and V.V. Yaglov: Conceived and designed the experiments; Performed the experiments; Analyzed and interpreted the data; Wrote the paper.

E.S. Tsomartova, M.Y. Ivanova, E.V. Chereshneva, S.G. Muhamedova and T.A. Lomanovskaya: Performed the experiments; Analyzed and interpreted the data; Wrote the paper.

### Funding statement

The research was financially supported by the Ministry of Science and Higher Education of the Russian Federation (No. AAAA-A17-117013050048-6).

### Competing interest statement

The authors declare no conflict of interest.

### Additional information

No additional information is available for this paper.
